# Impact of Stress Annealing on the Magnetization Process of Amorphous and Nanocrystalline Co-Based Microwires

**DOI:** 10.3390/ma12162644

**Published:** 2019-08-20

**Authors:** Ahmed Talaat, Valentina Zhukova, Mihail Ipatov, Juan María Blanco, Julián Gonzalez, Arcady Zhukov

**Affiliations:** 1 Departamento de Física de Materiales, Facultad de Químicas, Universidad del País Vasco/Euskal Herriko Unibersitatea, 20018 San Sebastian, Spain; 2 Departamento de Física Aplicada, Escuela Universitaria Politécnica de Donostia-San Sebastián, Universidad del País Vasco/Euskal Herriko Universitatea, 20018 San Sebastián, Spain; 3 IKERBASQUE, Basque Foundation for Science, 48011 Bilbao, Spain

**Keywords:** domain wall dynamics, stress annealing, magnetostriction, nanocrystalline microwires

## Abstract

The domain wall (DW) dynamics of amorphous and nanocrystalline Co-based glass-coated microwires are explored under the influence of stress annealing. Different annealing profiles have enabled remarkable changes in coercivity and magnetostriction values of Co-based amorphous microwires with initially negative magnitude, allowing induced magnetic bistability in stress-annealed samples and, consequently, high DW velocity has been observed. Similarly, Co-based nanocrystalline microwires with positive magnetostriction and spontaneous bistability have featured high DW velocity. Different values of tensile stresses applied during annealing have resulted in a redistribution of magnetoelastic anisotropy showing a decreasing trend in both DW velocities and coercivity of nanocrystalline samples. Observed results are discussed in terms of the stress dependence on magnetostriction and microstructural relaxation.

## 1. Introduction

Domain wall (DW) propagation has become a topic in the spate of a number of emerging applications including a wide range of magnetic logics and nano-sized data storage devices [1,2,3,4]. The dynamic behavior of magnetic DWs determines the operational speed of such devices, and thereby knowledge of such dynamics and their dependence on external stimuli (e.g., magnetic field, current, tensile stress, frequency) is crucial on both device and materials levels [3,4,5]. To this end, amorphous and nanocrystalline soft magnetic glass-coated microwires have attracted great attention as a result of their outstanding magnetic properties (with low coercivities down to 4 A/m) and production techniques allowing considerable diameter reductions in comparison with other rapidly quenched materials [6,7,8].

Glass-coated microwires are composite materials made of a metallic nucleus (amorphous alloy) covered by a glass-coating layer [7,9,10]. The variety of dimensions at the micro-scale, as well as different chemical compositions, are easily obtainable by the modified Taylor–Ulitovsky fabrication method based on rapid solidification phenomena [10]. The interaction of local magnetic moments with stresses arising from fast drawing, as well as different thermal expansion coefficients of the metallic nucleus and glass shell during a microwire’s production, defines the magnetoelastic anisotropy. Hence, the magnetoelastic anisotropy is regulated by

*K_me_* ≈ 3/2 *λ_s_ σ_internal_* (1)

where *λ_s_* is the saturation magnetostriction coefficient, and *σ_internal_* is the internal stresses induced during the fabrication process.

The sign and magnitude of the magnetostriction coefficient determines either the domain structure or hysteresis loop character of glass-coated microwires. In addition, in low magnetostrictive compositions, the stress dependence of magnetostriction either applied or internal can be relevant [11,12]. The dependence of the magnetostriction on stress is expressed as

*λ_s_* (*σ*) = *λ_s_* (0) − *Bσ* (2)

where λ_s_(σ) is the magnetostriction constant under stress; λ_s_(0) is the zero-stress magnetostriction constant; B is a positive coefficient of order 10^−10^ MPa, and σ is applied or internal stresses. This change of the magnetostriction can be associated with both applied, σ_applied_, and/or internal, σ_internal_, stresses (σ_total_ = σ_applied_ + σ_internal_). In accordance, for the low-magnetostrictive compositions (with λ_s_ (0) ≈ 10^−7^) and internal stresses of the order of 1000 MPa, the second term of Equation (2) is almost of the same order as the first one. Correspondingly, the magnetostriction is a key factor to tune magnetic properties of glass-coated microwires. 

The characteristic feature of amorphous glass-coated microwires with positive magnetostriction is the spontaneous magnetic bistability associated with a single-step axial magnetization reversal and fast DW propagation [6,13]. Negative and nearly zero magnetostrictive microwires, on the other hand, display an unhysteretic hysteresis loop and attractive high frequency magneto-transport properties (also known as the giant magnetoimpedance effect) [8,14,15].

Conventional annealing is a typical approach to improve the magnetic properties of magnetic materials (e.g., permalloy, Si steels, etc.) [16], and in particular of rapidly quenched soft magnetic alloys [17]. Accordingly, a few attempts to tailor magnetic properties of glass-coated microwires by conventional annealing (without stress) have been reported [18,19]. Surprisingly, considerable magnetic hardening has been observed upon annealing of Co-based microwires with a vanishing magnetostriction coefficient [18]. However, we recently observed that stress annealing can be an even more effective method for tuning the magnetic properties of glass-coated microwires [20]. In particular, we recently observed that stress annealing allows the induction of a transverse magnetic anisotropy that can be more effective for tuning the magnetic properties of glass-coated microwires. Therefore, the effect of annealing in the presence of applied stresses (stress annealing) is quite relevant and important to investigate.

Studies of DW dynamics in glass-coated microwires have been a subject of intensive research [21,22,23,24]. Achieving high DW velocities in these materials has relied on geometrical constraints (i.e., different values of internal stresses) and special magnetic structures formed in either positive, negative, or vanishing magnetostrictive compositions. In particular, around vanishing but still negative magnetostriction, fast DW velocities have been reported in amorphous Co-based microwires by means of controlling the annealing treatment conditions (i.e., changing the annealing temperature, time, and applied stresses) [18,25]. In these previous studies, the features of induced magnetic bistability have been discussed in consideration of the effect of annealing on the magnetostriction values and formation of axial domain structures in the inner cores of microwires. 

In this research, we report upon the influence of stress annealing on the magnetic properties and DW dynamics of Co-based amorphous and nanocrystalline glass-coated microwires with induced and spontaneous magnetic bistability, respectively. We evaluate the effect of different stress values on the magnetic properties and DW dynamics.

## 2. Materials and Methods 

**Figure 1:**
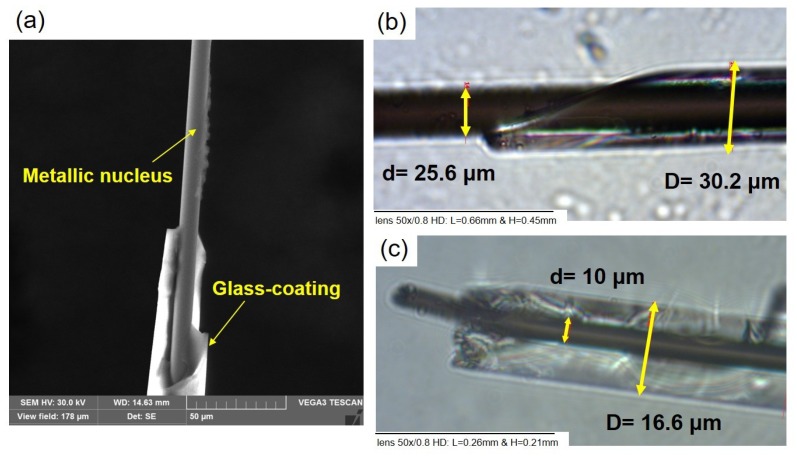
SEM image of a Co_38.5_Fe_38.5_B_18_Mo_4_Cu_1_ microwire (**a**) and optical microscope images in the transmitted light mode showing sample diameters for the (**b**) Co_69.2_Fe_4.1_B_11.8_Si_13.8_C_1.1_ and (**c**) Co_38.5_Fe_38.5_B_18_Mo_4_Cu_1_ microwires. The yellow arrows are for guidance.

Amorphous Co_69.2_Fe_4.1_B_11.8_Si_13.8_C_1.1_ (total diameter D ≈ 30.2 μm; metallic nucleus diameter d ≈ 25.6 μm) and nanocrystalline Co_38.5_Fe_38.5_B_18_Mo_4_Cu_1_ (total diameter D ≈ 16.6 μm; metallic nucleus diameter d ≈ 10 μm) glass-coated microwires have been prepared by means of a modified Taylor–Ulitovsky technique described elsewhere [9,10]. Scanning electron microscopy (SEM, TESCAN model VEGA, UPJS, Kosice, Slovakia) was used to examine the wires’ topologies (Figure 1a). The diameters of the metallic nucleus and the glass shell were determined using an optical microscope (Axio Scope A1, Carl Zeiss, Jena, Germany), as shown in Figure 1b,c.

Structure and phase compositions of as-prepared microwires were characterized by a BRUKER (D8 Advance, Karlsruhe, Germany) X-ray diffractometer with Cu-Kα (λ = 0.15406 nm) radiation. The samples were attached to the diffractometer, at which each scan was made over a two-theta angular range of 30–90 degrees with a step size of 0.05° and a step time of 30 seconds for each step.

We performed DW measurements with a set-up system consisting of three pickup coils based on the classic Sixtus–Tonks experiments [26]. Stress-annealed microwire samples (10 cm long) were placed coaxially inside of pickup coils. A magnetic field was generated by a solenoid upon applying rectangular-shaped voltage. As described previously [22,23,27], the DW propagation induces electromotive force (EMF) in the coil that is picked up at an oscilloscope upon passing the propagating wall. The DW velocity was estimated as

*υ = l/Δt* (3)

where *l* is the distance between pick-up coils and *Δt* is the time difference between the maximum in the induced EMF. In reported DW velocities, we excluded the non-linearity at high values of magnetic fields from the discussion and aimed only at the DW characteristics in the viscous regime.

Hysteresis loops of as-prepared and stress-annealed samples were measured by the flux metric methods used in [8]. We represented the hysteresis loops as normalized magnetization, M/Ms, versus the applied magnetic field, H, where Ms is the magnetic moment of the sample at the maximum magnetic field amplitude H_o_.

The stress-annealing process was carried out in a conventional furnace where a mechanical load was attached into one end of the microwire and axially placed via the furnace nozzle. Values of the applied stresses were calculated based on Young moduli of the metallic nuclei and the glass shell, as well as the microwire cross-sectional area, described in [25]. 

Magnetostriction coefficients were measured by the small-angle magnetization rotation (SMAR) methods described elsewhere [12,28]. Briefly, the magnetostriction coefficient was evaluated according to dependence of the axial magnetic field on an applied stress at the fixed value of the induction voltage *V(*2*f)*, according to the expression

*λ_s_ = − (μ_ο_M_s_/*3*)(ΔH/Δσ_app_)_V(*2*f)_ = constant* (4)

where *μ_ο_M_s_* is the saturation magnetization, and *σ_app_* is the applied tensile stress.

## 3. Results and Discussion 

**Figure 2:**
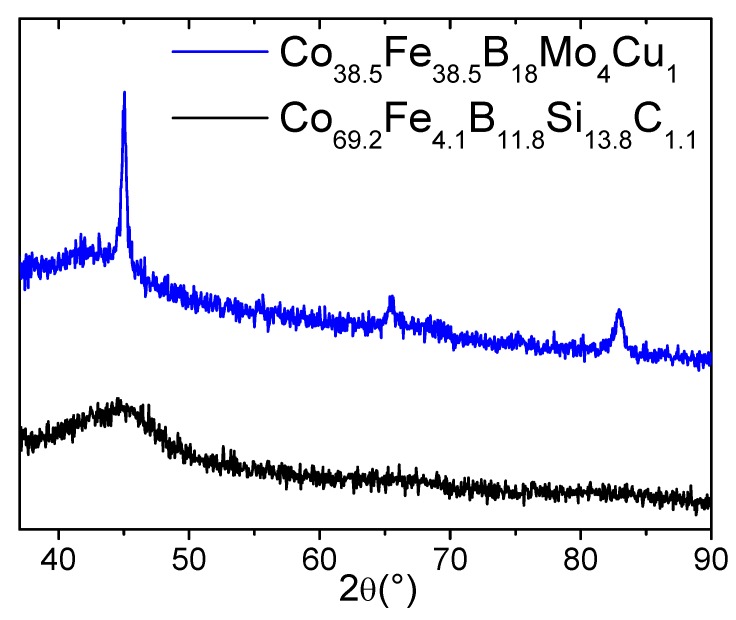
XRD patterns of as-prepared Co_69.2_Fe_4.1_B_11.8_Si_13.8_C_1.1_ and Co_38.5_Fe_38.5_B_18_Mo_4_Cu_1_ glass-coated microwires.

Figure 2 shows XRD patterns of as-prepared Co_69.2_Fe_4.1_B_11.8_Si_13.8_C_1.1_ and Co_38.5_Fe_38.5_B_18_Mo_4_Cu_1_ glass-coated microwires. As can be clearly seen, the Co_69.2_Fe_4.1_B_11.8_Si_13.8_C_1.1_ microwires presented an amorphous structure confirmed by a diffuse halo, without observation of any crystalline peaks. In contrast, notable crystalline peaks corresponding with α-FeCo phase (33 nm average grain size) were shown for Co_38.5_Fe_38.5_B_18_Mo_4_Cu_1_ microwires. It is worth underlining that, in some cases, decreasing the quenching speed during the fabrication process resulted in formation of nanocrystallites embedded within an amorphous matrix. Analysis of the crystalline peak features and average grain size calculation have been reported earlier by us for different Fe- and Co-based of nanocrystalline microwires [29].

**Figure 3:**
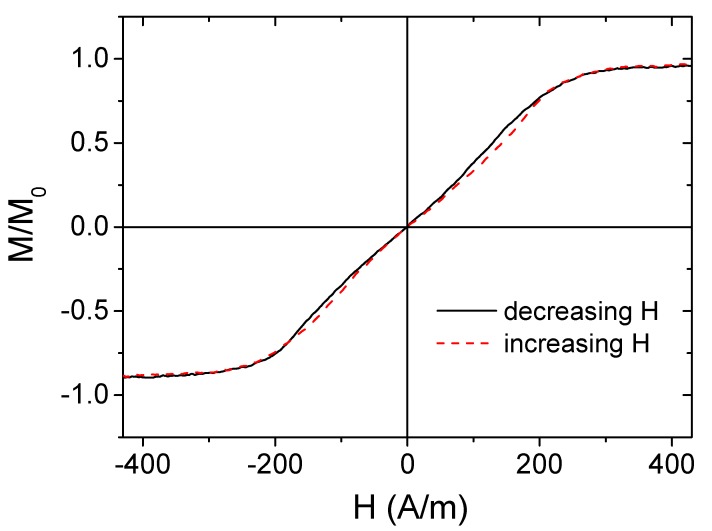
Hysteresis loop of as-prepared Co_69.2_Fe_4.1_B_11.8_Si_13.8_C_1.1_ microwires.

As-prepared amorphous Co_69.2_Fe_4.1_B_11.8_Si_13.8_C_1.1_ microwires presented an almost unhysteretic shape with low coercivity, H_c_≈ 4A/m (Figure 3), as previously reported for Co-based glass-coated microwires with low and negative λ_s_ (λs ≈ −10^−7^). Slightly irregular hysteresis loop shapes can be related either to the interface layer between the metallic nucleus and glass coating [30], or to a contribution of the small inner axially magnetized domain that usually presents higher coercivity.

**Figure 4:**
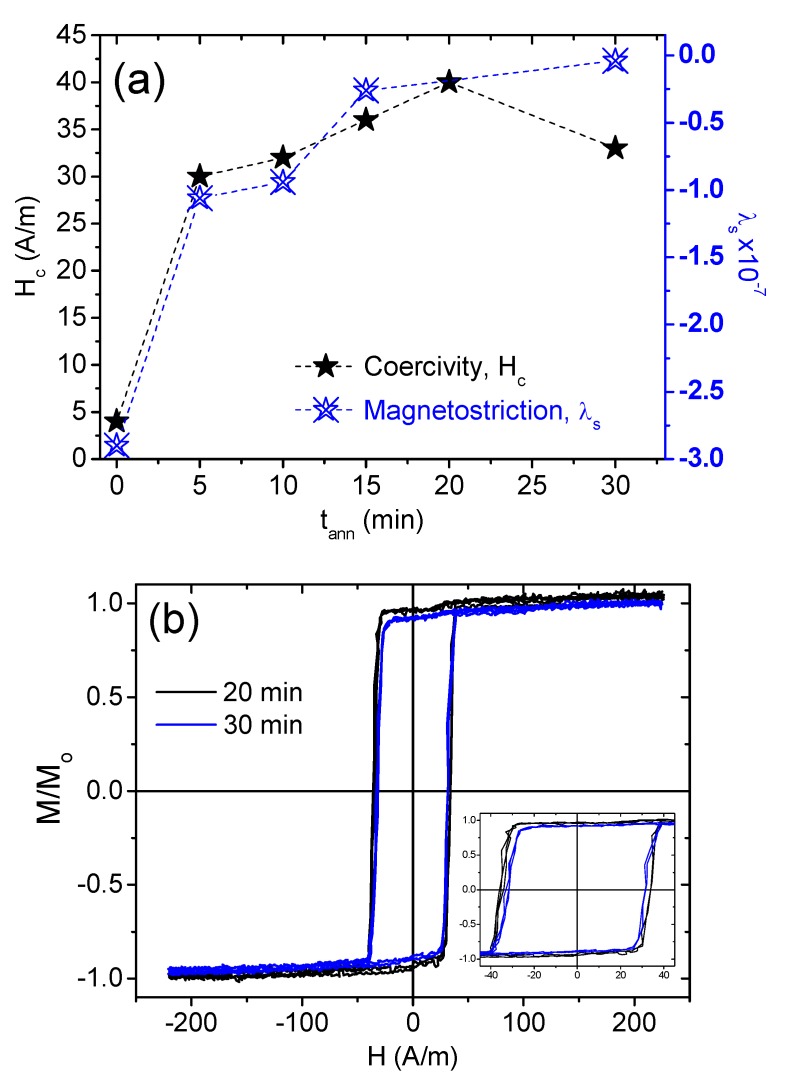
Evaluation of the coercivity and magnetostriction coefficient of stress-annealed Co_69.2_Fe_4.1_B_11.8_Si_13.8_C_1.1_ microwires versus annealing time at a fixed annealing temperature of 300 °C with an 80-MPa applied stress (**a**), and hysteresis loops of stress-annealed samples for 20 and 30 min (**b**).

For Co_69.2_Fe_4.1_B_11.8_Si_13.8_C_1.1_ amorphous samples, we performed a series of stress-annealing steps varying the annealing time from 5 to 30 min at a fixed annealing temperature (300 °C) and 80 MPa applied stress. The evaluation of coercivity as well as magnetostriction with annealing time are plotted in Figure 4a. As can be appreciated, stress annealing resulted in an increase of coercivity (Hc increased from 4 A/m in as-prepared samples to 33 A/m in stress-annealed samples at t_ann_ 30 min). In most cases, conventional annealing methods result in decreasing coercivity due to internal stress relaxation induced by the fabrication process. However, in the case of Co-based microwires, considerable magnetic hardening upon annealing has been recently observed [18]. In the present case, observed Hc values were considerably lower than those reported in [18]. Such magnetic hardening can be explained by considering the effect of annealing on the magnetostriction coefficient.

Consequently, we evaluated the λ_s_ values upon stress annealing. The magnetostriction shifted from highly negative to nearly zero in as-prepared versus stress-annealed Co_69.2_Fe_4.1_B_11.8_Si_13.8_C_1.1_ microwire samples at t_ann_ = 30 min, respectively. In the present case of stress annealing (i.e., conventional annealing simultaneously under tensile stress) the situation was rather complex. 

As such, two different induced anisotropies were present: one arising from the mechanical load (transversal), and the other from annealing (stress relaxation and hence magnetostriction modification), leading to a redistribution of the internal stresses and/or local microstructures of the sample. In addition, if the initial magnetostriction coefficient is low and negative, we must consider two opposite consequences on the internal stresses. The first contribution is an increase of the total magnetoelastic energy. The second must be related to stress dependence (either applied or internal stresses: σ_total_ = σ_applied_ + σ_internal_) on the magnetostriction coefficient, described in Equation (2), which is quite relevant in the case of a low magnetostriction constant, λ_s,0_. 

**Figure 5:**
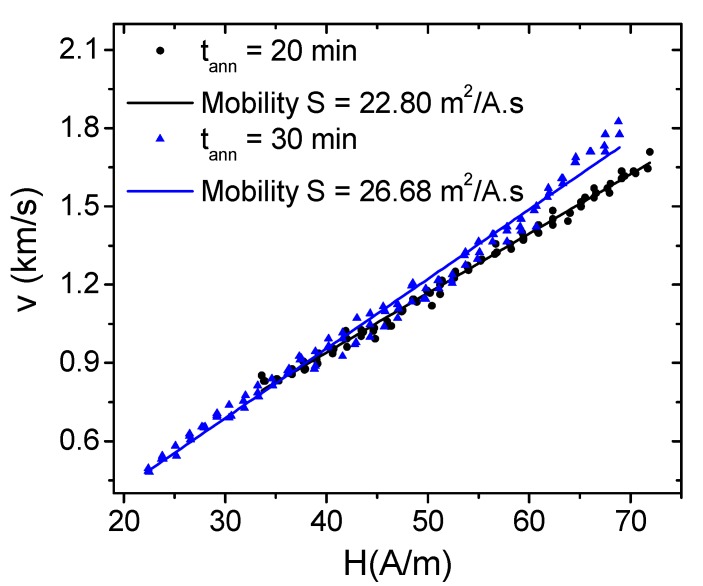
Domain wall velocity and calculated mobility for stress-annealed Co_69.2_Fe_4.1_B_11.8_Si_13.8_C_1.1_ microwires performed at a fixed annealing temperature of T_ann_ = 300 °C under 80 MPa for t_ann_ = 20 and 30 min.

We analyzed DW dynamics in consideration of the observed rectangular character of hysteresis loops and induced magnetic bistability of stress-annealed Co_69.2_Fe_4.1_B_11.8_Si_13.8_C_1.1_ microwire (presented in Figure 4b). Due to a rectangular hysteresis loop, the remagnetization of this sample ran through the DW propagation within an inner single-domain core [18,25]. As can be clearly seen in Figure 5, the DW velocity dependencies on applied magnetic fields presented typical DW linear growth velocity with the magnetic field.

The response of a DW to a magnetic field in a viscous medium is described by the classical equation of motion [31]. This leads to the following expression for the steady-state wall velocity:

*υ = S (H − H_0_)* (5)

where *H* is the axial magnetic field, *H_0_* is the critical propagation field below which DW propagation is not possible, and *S* is the DW mobility regulated by S ≈ δ ≈ (A/K_me_)^1/2^, where A is the exchange stiffness constant and K_me_ (Equation (1)) is the magnetic anisotropy constant. Consequently, both DW velocity and mobility are strongly dependent on magnetoelastic anisotropy. Reasonably high DW velocities and DW mobilities are both shown in Figure 5. In parallel to the hysteresis loops presented in Figure 4b, stress-annealed samples for t_ann_ = 30 min showed either higher DW velocity or mobility than those annealed for t_ann_ = 20 min. In particular, DW mobility increased from 22.80 m^2^/A.s for samples stress annealed for 20 min, to 26.68 m^2^/A.s after 30 min annealing. Higher DW mobility should be ascribed to lower magnetoelastic anisotropy, deduced from the low coercivity, as well as the near-zero magnetostriction, which is shown in Figure 4.

**Figure 6:**
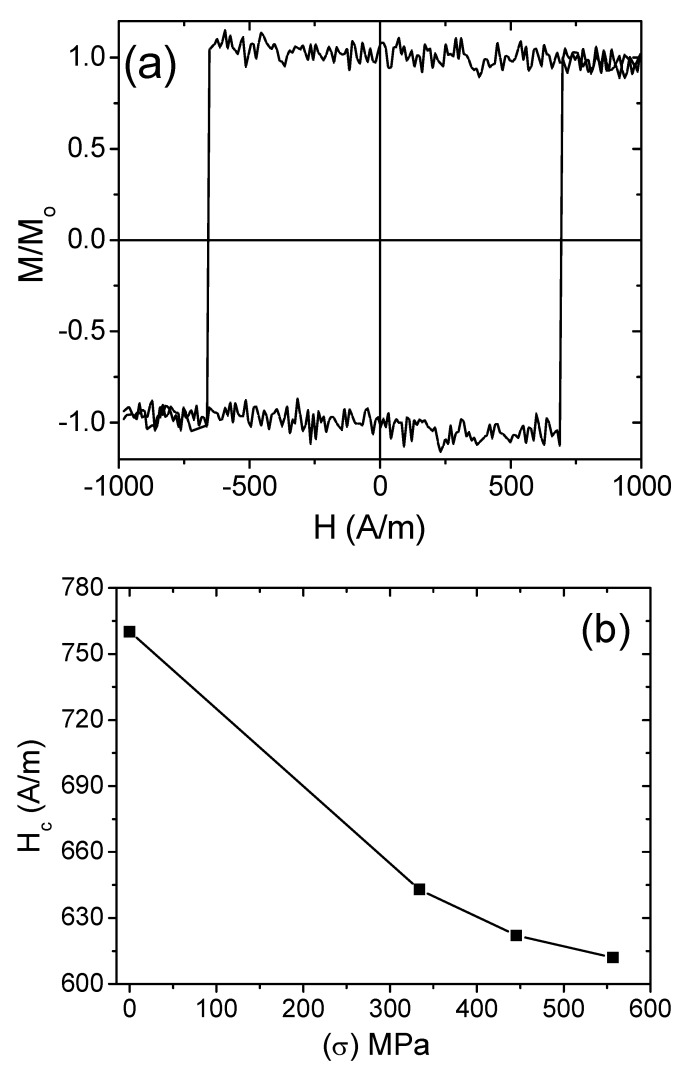
Hysteresis loops of as-prepared Co_38.5_Fe_38.5_B_18_Mo_4_Cu_1_ microwires (**a**), and coercivity dependence on applied stresses of stress-annealed Co_38.5_Fe_38.5_B_18_Mo_4_Cu_1_ microwires at an annealing temperature of 300 °C for 1 h (**b**).

**Figure 7:**
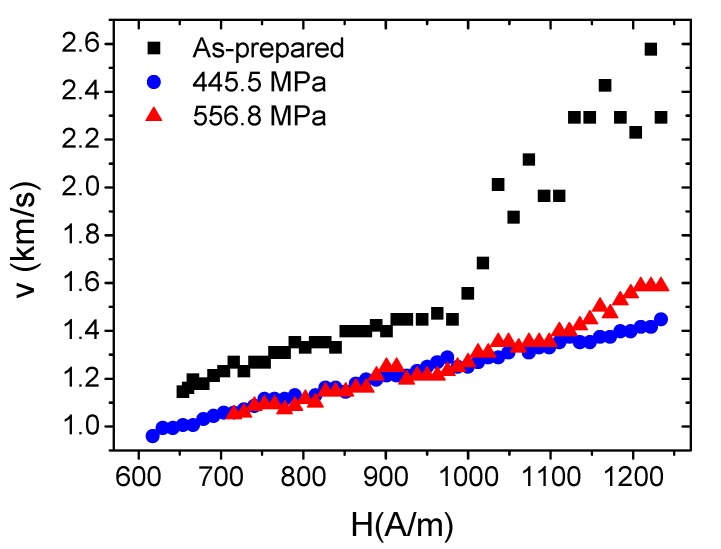
Domain wall velocity of as-prepared and stress-annealed Co_38.5_Fe_38.5_B_18_Mo_4_Cu_1_ microwires at 300 °C for 1 h under different values of applied stresses.

On the other hand, as-prepared Co_38.5_Fe_38.5_B_18_Mo_4_Cu_1_ nanocrystalline microwires presented higher values of coercivity and rectangular hysteresis loops with spontaneous magnetic bistability, as shown in Figure 6a. Stress annealing resulted in a considerable decrease of coercivity; the coercivity decreased from 760 A/m in as-prepared samples to 610 A/m in stress-annealed samples at 300 °C for 1 h under 556.8 MPa applied stress. The dependence of coercivity on different values of applied stresses during annealing is presented in Figure 6b. As can be appreciated, a decreasing trend of coercivity after stress annealing is observed. This is due to the compressive and back stresses induced by the mechanical loads during annealing as well as the glass shell (i.e., upon removing the mechanical loads, compressive or back stresses evolved) as reported previously for Fe-based amorphous microwires [32]. A similar tendency of the DW velocity dependence on magnetic fields of stress-annealed Co_38.5_Fe_38.5_B_18_Mo_4_Cu_1_ microwires was also observed, as shown in Figure 7.

As shown, DW velocities decreased upon stress annealing. It is well known that in nanocrystalline alloys, the internal stresses are heterogeneously distributed into two phases (i.e., an amorphous phase and a nanocrystalline phase). As reported previously [33,34], the easy magnetization axis in nanocrystalline alloys can be either parallel or perpendicular to the direction of stresses applied during annealing, depending on the sign of the magnetostriction and chemical alloy compositions. Accordingly, Equation (2) can be reproduced as

(6) $$K_{\mathrm{me}}\approx 3/2\;(V_{cr}\;\lambda_{s}^{Cr}\sigma_{cr}\;+\;\left(1-\;V_{cr}\right)\;\lambda_{s}^{am}\;\sigma_{am})$$

where V_cr_ is crystalline volume fraction, and σ_cr_ and σ_am_ denote the stresses located in the volume fraction of nanocrystallites and the amorphous phase, respectively.

In the case of Fe-based nanocrystalline microwires (for example Finemet alloys), negative magnetostriction of nanocrystallites (α-FeSi) compensate for the positive magnetostriction of the parent amorphous phase yielding to vanishing magnetostriction values and overall low magnetoelastic anisotropy. The application of tensile stresses during annealing of Finemet alloys develops inclined hysteresis loops with an easy magnetization axis that is perpendicular to the direction of the tensile stress [34]. In contrast, α-FeCo nanocrystallites have been observed with positive magnetostriction in Co_38.5_Fe_38.5_B_18_Mo_4_Cu_1_ nanocrystalline microwires, and for that reason overall magnetostriction has remained positive, with rectangular hysteresis loops. The character of such hysteresis loops preserves rectangular with spontaneous magnetic bistability even after annealing (cf. Figure 6 in [35]). Consequently, it can be deduced that the easy magnetization axis is parallel to the direction of stresses. As such, the overall magnetoelastic anisotropy is reasonably higher (according to Equation (6)), resulting finally in the lower DW dynamics observed in Figure 7. The DW mobility decreased from 0.97 m^2^/A.s in as-prepared Co_38.5_Fe_38.5_B_18_Mo_4_Cu_1_ nanocrystalline samples to 0.71 m^2^/A.s in stress-annealed samples at 556.8 MPa.

## 4. Conclusions

In summary, we have evaluated different stress-annealing conditions to study the magnetization process and DW dynamics of Co-based amorphous and nanocrystalline glass-coated microwires. Stress annealing of amorphous Co_69.2_Fe_4.1_B_11.8_Si_13.8_C_1.1_ microwires resulted in increasing coercivity with induced magnetic bistability and considerable changes in magnetostriction. The latter was elevated from highly negative to vanishing values. Fast DW velocities and mobilities appeared with unusual features in stress-annealed amorphous microwires. The opposite tendency of DW dynamics was observed in the case of nanocrystalline Co_38.5_Fe_38.5_B_18_Mo_4_Cu_1_ microwires with highly positive magnetostriction. Induced magnetoelastic anisotropy upon increasing applied stresses during annealing resulted in decreasing the coercivity and hence lowering DW dynamics.

## References

[1] Atkinson D., Allwood D.A., Xiong G., Cooke M.D., Faulkner C.C., Cowburn R.P. (2003). Magnetic domain-wall dynamics in a submicrometre ferromagnetic structure. Nat. Mater..

[2] Allwood D.A., Xiong G., Faulkner C.C., Atkinson D., Petit D., Cowburn R.P. (2005). Magnetic Domain-Wall Logic. Science.

[3] Beach G.S.D., Nistor C., Knutson C., Tsoi M., Erskine J.L. (2005). Dynamics of field-driven domain-wall propagation in ferromagnetic nanowires. Nat. Mater..

[4] Parkin S.S.P., Hayashi M., Thomas L. (2008). Magnetic Domain-Wall Racetrack Memory. Science.

[5] Estévez V., Laurson L. (2015). Head-to-head domain wall structures in wide permalloy strips. Phys. Rev. B.

[6] Vázquez M., Chiriac H., Zhukov A., Panina L., Uchiyama T. (2011). On the state-of-the-art in magnetic microwires and expected trends for scientific and technological studies. Phys. Status Solidi A.

[7] Zhukov A., Ipatov M., Talaat A., Blanco J.M., Hernando B., Gonzalez-Legarreta L., Suñol J.J., Zhukova V. (2017). Correlation of Crystalline Structure with Magnetic and Transport Properties of Glass-Coated Microwires. Crystals.

[8] Zhukov A., Ipatov M., Churyukanova M., Talaat A., Blanco J.M., Zhukova V. (2017). Trends in optimization of giant magnetoimpedance effect in amorphous and nanocrystalline materials. J. Alloys Compd..

[9] Chiriac H., Óvári T.A. (1996). Amorphous glass-covered magnetic wires: Preparation, properties, applications. Prog. Mater. Sci..

[10] Larin V.S., Torcunov A.V., Zhukov A., González J., Vazquez M., Panina L. (2002). Preparation and properties of glass-coated microwires. J. Magn. Magn. Mater..

[11] Barandiarán J.M., Hernando A., Madurga V., Nielsen O.V., Vázquez M., Vázquez-López M. (1987). Temperature, stress, and structural-relaxation dependence of the magnetostriction in (Co_0.94_Fe_0.06_)_75_Si_15_B_10_ glasses. Phys. Rev. B.

[12] Zhukov A., Churyukanova M., Kaloshkin S., Sudarchikova V., Gudoshnikov S., Ipatov M., Talaat A., Blanco J.M., Zhukova V. (2016). Magnetostriction of Co-Fe-Based Amorphous Soft Magnetic Microwires. J. Electron. Mater..

[13] Varga R., García K.L., Vázquez M., Zhukov A., Vojtanik P. (2004). Switching-field distribution in amorphous magnetic bistable microwires. Phys. Rev. B.

[14] Phan M.-H., Peng H.-X. (2008). Giant magnetoimpedance materials: Fundamentals and applications. Prog. Mater. Sci..

[15] Zhukov A., Ipatov M., Zhukova V., Buschow K.H.J. (2015). Chapter 2—Advances in Giant Magnetoimpedance of Materials. Handbook of Magnetic Materials.

[16] Bozorth R.M. (1953). The Permalloy Problem. Rev. Mod. Phys..

[17] Herzer G. (2013). Modern soft magnets: Amorphous and nanocrystalline materials. Acta Mater..

[18] Zhukov A., Talaat A., Ipatov M., Blanco J.M., Zhukova V. (2014). Tailoring of magnetic properties and GMI effect of Co-rich amorphous microwires by heat treatment. J. Alloys Compd..

[19] Zhukova V., Talaat A., Ipatov M., Blanco J.M., Phan M., Zhukov A.P. (2014). Effect of Annealing on Magnetic Properties and Giant Magnetoimpedance Effect of Amorphous Microwires. IEEE Trans. Magn..

[20] Zhukova V., Blanco J.M., Ipatov M., Churyukanova M., Taskaev S., Zhukov A. (2018). Tailoring of magnetoimpedance effect and magnetic softness of Fe-rich glass-coated microwires by stress- annealing. Sci. Rep..

[21] Jiménez A., del Real R.P., Vázquez M. (2013). Controlling depinning and propagation of single domain-walls in magnetic microwires. Eur. Phys. J. B.

[22] Zhukov A., Blanco J.M., Ipatov M., Talaat A., Zhukova V. (2017). Engineering of domain wall dynamics in amorphous microwires by annealing. J. Alloys Compd..

[23] Klein P., Varga R., Vázquez M. (2013). Stable and fast domain wall dynamics in nanocrystalline magnetic microwire. J. Alloys Compd..

[24] Beck F., Rigue J.N., Carara M. (2017). The profile of the domain walls in amorphous glass-covered microwires. J. Magn. Magn. Mater..

[25] Talaat A., Blanco J.M., Ipatov M., Zhukova V., Zhukov A.P. (2014). Domain Wall Propagation in Co-Based Glass-Coated Microwires: Effect of Stress Annealing and Tensile Applied Stresses. IEEE Trans. Magn..

[26] Sixtus K.J., Tonks L. (1932). Propagation of Large Barkhausen Discontinuities. II. Phys. Rev..

[27] Zhukova V., Blanco J.M., Rodionova V., Ipatov M., Zhukov A. (2012). Domain wall propagation in micrometric wires: Limits of single domain wall regime. J. Appl. Phys..

[28] Narita K., Yamasaki J., Fukunaga H. (1980). Measurement of saturation magnetostriction of a thin amorphous ribbon by means of small-angle magnetization rotation. IEEE Trans. Magn..

[29] Talaat A., del Val J.J., Zhukova V., Ipatov M., Klein P., Varga R., González J., Churyukanova M., Zhukov A. (2016). Grain size refinement in nanocrystalline Hitperm-type glass-coated microwires. J. Magn. Magn. Mater..

[30] Zhukov A., Shuvaeva E., Kaloshkin S., Churyukanova M., Kostitsyna E., Zhdanova M., Talaat A., Ipatov M., Zhukova V. (2016). Studies of Interfacial Layer and Its Effect on Magnetic Properties of Glass-Coated Microwires. J. Electron. Mater..

[31] O’Handley R.C. (1975). Domain wall kinetics in soft ferromagnetic metallic glasses. J. Appl. Phys..

[32] Zhukov A. (2006). Design of the Magnetic Properties of Fe-Rich, Glass-Coated Microwires for Technical Applications. Adv. Funct. Mater..

[33] Alves F., Desmoulins J.B., Hérisson D., Rialland J.F., Costa F. (2000). Stress-induced anisotropy in Finemet- and Nanoperm-type nanocrystalline alloys using flash annealing. J. Magn. Magn. Mater..

[34] Herzer G., Budinsky V., Polak C. (2011). Magnetic properties of FeCuNbSiB nanocrystallized by flash annealing under high tensile stress. Phys. Status Solidi B.

[35] Talaat A., Del Val J.J., Zhukova V., Ipatov M., Klein P., Varga R., Gonzalez J., Zhdanova M., Churyukanova M., Zhukov A. (2016). Effect of annealing on magnetic properties of nanocrystalline Hitperm-type glass-coated microwires. J. Alloys Compd..

